# Regional Entrepreneurship, Business Environment, and High-Quality Economic Development: An Empirical Analysis of Nine Urban Agglomerations in China

**DOI:** 10.3389/fpsyg.2022.905590

**Published:** 2022-05-17

**Authors:** Ce Guo, Chao Liu, Qiwei Xie, Xiaole Lin

**Affiliations:** ^1^College of Economics and Management, Beijing University of Technology, Beijing, China; ^2^College of Finance and Economics, Shandong University of Science and Technology, Tai’an, China

**Keywords:** business environment, regional entrepreneurship, system dynamics, city cluster, high-quality economic development

## Abstract

The article selects socioeconomic data related to 146 prefecture-level cities included in nine city clusters from 2014 to 2018 to establish a city-level socioeconomic system in China. A sensitivity analysis of regional entrepreneurship and economic quality development based on system dynamics was conducted to explore the changes in regional entrepreneurship and economic quality development over time and their sensitivity factors. In this way, the dynamic evolution mechanism of the system can be portrayed, and the optimization of the system can be achieved through the coordination of the factors within the system. The article sets up three scenarios to explore the fluctuations in regional entrepreneurship and economic quality development when three sensitive factors, namely, business environment, financial services scale, and innovation environment, change. Findings: There are differences in the development of cities within city clusters. The business environment and high-quality economic development of the central cities within the city cluster are stronger than those of the non-central cities. Therefore, regions should focus on synergistic development within city clusters when formulating related policies. The variation of regional entrepreneurship development and economic quality development, after a factor in the system is changed, is asymmetric. Because the sensitivity of different urban clusters and the way they are affected by sensitive factors varies, the state should pay more attention to the adaptability of cities when formulating corresponding policy measures and adapt its policy measures to the sensitivity characteristics of each region according to local conditions.

## Introduction

A good business environment will promote entrepreneurship, an essential driver of economic growth, and can be a new engine for China’s high-quality economic development. Since the reform and opening up in 1978, the regions have made efforts to develop their economies, but the current situation shows a clear gap between the economic conditions of the different regions. The reasons can be divided into two main aspects: historical and practical. The historical reason is that the strategic concept of “two overall situations” was proposed during the reform and opening-up period. The state formulated a series of preferential policies to encourage entrepreneurship and innovation to promote the economic development of the eastern coastal region. The central and western provinces imported a steady flow of capital, natural resources, and human resources into the southeastern coastal region (Du et al., 2020). The regional entrepreneurial environment is uneven. Therefore, there is a certain historical inevitability to the differences in development between regions (Suddaby et al., 2015). The reality is that today’s market economy has been established, and competition in the market has increased. Even with the call for “mass entrepreneurship and innovation,” there is still a wide gap in regional economic development. The unstable business environment has become uneven. Every city wants to solve the urgent problem of how to improve the business environment to promote regional entrepreneurship and enhance the quality of economic development. Currently, urban agglomerations are a new regional unit enabling countries to participate in global competition and division of labor (Porter, 2000).

In this paper, we take economic development issues as a guide and use urban agglomerations to explore regional entrepreneurship and economic quality development sensitivity factors and optimization issues. First, the CRITIC empowerment method is applied to evaluate the quality of the business environment and economic development of the selected cities and urban clusters. Then, we construct a Chinese socioeconomic system and conduct a sensitivity analysis of regional entrepreneurship and quality economic development based on system dynamics. In this way, strategic solutions to enhance regional entrepreneurship and economic development are explored.

After the introduction, the rest of the paper is structured as follows: The second section is the literature review. The third section is measures, describing the data sources and indicators. The fourth section sets out China’s city-level socioeconomic system and establishes a cause-and-effect diagram of each sub-system and the total system, aggregated to form a structural flow diagram of the total system. The fifth section presents the results and findings of this research. First, the business environment and the quality of economic development are evaluated. Then, simulation predictions and sensitivity analysis based on system dynamics explore the impact on regional entrepreneurship and high-quality economic development when the business environment, financial services scale, and innovation environment change. The sixth section contains the discussion and conclusions.

## Literature Review

Optimizing the business environment is a meaningful way to achieve high-quality development from high growth. Scholars view the business environment as an institutional policy environment and quantify its essential role in entrepreneurship and economic development (Davari et al., 2018). On the national level, since the implementation of China’s reform and opening up, the economic system has been reformed continuously to promote innovation and entrepreneurial development taking China’s economic development to an unprecedented level and promoting continuous rapid growth (Bradley et al., 2021). At the enterprise level, the optimization of the business environment is mainly reflected in the reduction in the tax burden on companies and the increase in companies’ property rights protection. These factors reduce companies’ transaction costs and transaction risks (Simmons, 2006; Acemoglu et al., 2015; Gailmard, 2020; Wang and Han, 2021). Fabro and Aixalá (2009) found that soft environment improvements to the business system implemented for SMEs can boost regional economic development.

Many factors affect the development of entrepreneurship (Castaño et al., 2015; Aparicio et al., 2016; Tur-Porcar et al., 2018; Cervelló-Royo et al., 2020; Méndez-Picazo et al., 2021). Many scholars have studied entrepreneurial development depending on the business environment to increase the number of entrepreneurs and the rate of business growth (Sattari and Mehrabi, 2016; Khan et al., 2019; Radović-Marković et al., 2019; Jang et al., 2020). Other researchers have explored the positive effects of financial development on promoting entrepreneurship (Civera et al., 2017; Léon, 2019; Hommel and Bican, 2020; Liu et al., 2020; Srivastava et al., 2021; Charfeddine and Zaouali, 2022). In addition, the development of innovation has contributed significantly to entrepreneurship and economic growth (Huggins and Thompson, 2015; Ferreira et al., 2017; Pounder, 2019; Pradhan et al., 2020).

The paradigm and methodology of systems science research can be adapted to the requirements of complex systems. As a powerful tool for system reflection, the system dynamics approach is a method for investigating, analyzing, and predicting system behavior and overcoming complexity (Hosseini and Shakouri, 2016). Jamshidi et al. (2021) applied system dynamics to identify strategies for the growth and development of start-up businesses, simulating current and future growth decisions, developing different scenarios, and proposing optimal policies. Their research provides a feasible method for the study of system dynamics in entrepreneurship. However, their study is based primarily on the operational level of the firm rather than on external economic factors.

Compared with the established literature, two possible innovative points in this paper are as follows: First, the article explores the mechanism of the role of the business environment, regional entrepreneurship, and high-quality economic development based on the perspective of system science. This paper applies a system dynamics approach to simulate the operation of a socioeconomic system based on the formation of complex interactions between factors within the system and predicting the future development of the system. In this way, the dynamic evolution mechanism of the system can be portrayed, and the optimization of the system can be achieved through the coordination of the factors within the system. Second, this article builds a city-level economic quality development evaluation index system. This paper draws on the evaluation system of provincial-level economic high-quality development constructed by Shi and Ren (2018) and Ou et al. (2020). Based on the five aspects of the new development concept, the key elements of high-quality economic development of cities and the differences between them and provincial elements are considered comprehensively to construct an evaluation index system for high-quality economic development at the city level.

## Measures

### Selection of Urban Agglomerations and Cities

Based on the classification of China’s urban agglomerations in 2018 and the six key regions of China mentioned by Li (2021), the study selected socioeconomic data related to 146 Chinese cities at prefecture level and above contained in nine urban agglomerations (seven national-level urban agglomerations and two provincial urban agglomerations). The nine urban agglomerations are Guangdong-Hong Kong-Macao, Yangtze River Delta, Central-Southern Area of Liaoning, Shandong Peninsula, Harbin-Changchun, Beijing-Tianjin-Hebei, Middle Yangtze, Chengdu-Chongqing, and the Central Plains urban agglomerations.

The data are mainly from the China Urban and Rural Construction Database, the China Urban Database, and the Urban Statistical Yearbook. Due to missing data, the Ha-Chang city cluster lacks data for the Yanbian Korean Autonomous Prefecture. The Middle Yangtze River city cluster lacks data for three cities: Xiantao, Qianjiang, and Tianmen. The Guangdong-Hong Kong-Macao Greater Bay Area lacks data for two cities, Hong Kong and Macau.

### Indicator Selection and Indicator System Construction

#### Regional Entrepreneurship Indicator Selection

The article draws on Tian and Chen (2016), who use the annual number of registered or newly established businesses to measure regional entrepreneurship. The data are taken from the China Basic Unit Statistical Yearbook and the China Urban Innovation and Entrepreneurship Index urban new business entry values.

#### Business Environment Indicator System Construction

The specific indicators of the indicator system of the business environment are mainly referred to the “Research on Evaluation of Doing Business in Chinese Cities” Research Group (2021) and the Project Team on “China’s Urban Business Environment Assessment and Research” From the Management World Economic Research Institute (2019) evaluation indicator system. Table 1 shows the business environment index system.

**Table 1:** Business environment indicator system.

	**Primary indicators**	**Secondary indicators**	**Tertiary indicators (Unit)**
Business environment	Government efficiency	Government payments	General budgetary expenditure (million)
		Government services	Governmental efficiency (%)
	Human resources	Labor costs	Average wage level (yuan)
		Human resource	Student enrollment (per person)
			Unit practitioners (per person)
	Financial services	Scale of practice	Financial practitioner (per 10,000 people)
		Financing services	The scale of private financing (per 10,000 RMB)
			Overall financing scale (per 10,000 RMB)
	Public service level	Gas supply capacity	Natural gas supply (million tons)
		Water supply	Water supply (million square meters)
		Electricity supply	Industrial electricity (million kWh)
		Medical conditions	Number of hospital beds (per million people)
	Market circumstances	Economic indicators	*Per capita* GDP (yuan)
			Fixed-asset investment (per 10,000 RMB)
		Import and export	Amount of foreign capital used (per 10,000 RMB)
			Number of new contracts signed
		Corporate institutions	Number of industrial enterprises above designated size
	Innovative environment	Innovative inputs	Scientific expenditure (per 10,000 RMB)
		Innovative outputs	Number of patents granted
		Innovation capability	Innovation capability index

#### System of Indicators for Quality Economic Development

The setting of the economic quality development index system is based on the five development concepts of the new development concept: innovation, coordination, green, openness, and sharing, mainly drawing on the Chinese provincial economic quality evaluation system constructed by Shi and Ren (2018) and Ou et al. (2020). The key elements of high-quality economic development in cities and the differences between them and the provincial elements are considered comprehensively to construct an evaluation index system for high-quality economic development at the city level in China. Table 2 shows the indicator system of high-quality economic development.

**Table 2:** System of indicators for quality economic development.

	**Primary indicators**	**Secondary indicators**	**Tertiary indicators (Unit)**
High-quality economic development	Innovation	Innovation inputs	Science and technology expenditure/GDP (%)
		Innovative outputs	Number of patents granted/total population (%)
	Coordination	Level of industry coordination	Tertiary industry/GDP (%)
		The urbanization rate	Urban population/Total population (%)
		Urban and rural income harmonized level	Disposable income per rural resident/Disposable income per urban resident (%)
	Green	Particulate emissions	Smoke (dust) emissions/GDP (%)
		Wastewater discharge	Wastewater discharge/GDP (%)
		Exhaust emission	Sulfur dioxide emissions/GDP (%)
	Openness	Import and export scale	Total imports and exports/GDP (%)
		Foreign trade dependence	Total foreign investment/GDP (%)
	Shared	Economic level	GDP per capita (RMB ten thousand/person)
		Educational situation	Education spending per capita (Yuan/person)
		Medical Services	Number of hospital beds per capita (per million people)

## Methodology

It is possible to cognize systems based on revealed laws and optimize the regulation of systems based on cognitive systems through simulation techniques. Only by studying and understanding the relationship between changes in the business environment that drive regional entrepreneurship and high-quality economic development from a systemic perspective can we accurately grasp the inherent laws of development as a whole. The business environment, regional entrepreneurship, and high-quality economic development are contained in a complex multi-layered socioeconomic system. To better describe the socioeconomic system, this study first decomposes it, constructs the sub-systems, and finally synthesizes them. Therefore, the sub-system is constructed on four levels: innovation of science and technology, economic development, public services and resources, and the environment. The following cause-effect diagrams and structural flow diagrams were drawn using Vensim software.

### Science and Technology Innovation Sub-system Construction

Adherence to the technology and innovation sub-system is the fundamental driving force for promoting economic development and improving entrepreneurship. The increased innovation capacity not only drives the development of the regional business environment and improves the core competitiveness of the region concerned but also reduces regional environmental pollution and promotes the development of a green economy. The science and technology innovation sub-system will be studied in the areas of innovation input, innovation output, and innovation capacity evaluation. It contains variables such as science and technology expenditure, number of patents granted, and innovation capacity. These variables also have a circular feedback relationship with the resource environment sub-system changes. The construction of this sub-system lays the foundation for the construction of the socioeconomic system.

**Figure 1:**
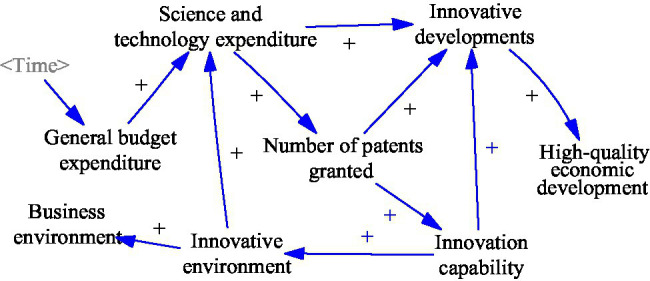
Technology and innovative sub-system causality diagram.

The main causal feedback loops of the technology and innovation sub-system are as follows, with the specific causal relationships shown in Figure 1.

1. General budget expenditure (+) → Science and technology expenditure (+) → Number of patents granted (+) → Innovative developments (+) → High-quality economic development (+)
2. General budget expenditure (+) → Science and technology expenditure (+) → Number of patents granted (+) → Innovation capability (+) → Innovative environment (+) → Business Environment (+).

### Economic Development Sub-system Construction

Achieving economic development is the primary goal of increasing entrepreneurship in the region, and the economic development sub-system is an essential component of a complex socioeconomic system. The economic development sub-system is studied in terms of openness to the outside world, coordinated urban–rural and industrial development, the efficiency of government services, human resources, and financial services. It contains variables such as investment in fixed assets, financial industry employees, and general budget expenditure. This sub-system is linked to the innovation and entrepreneurship sub-system, the public services sub-system, and the resources and environment sub-system. As the largest system in the socioeconomic system, the construction of this sub-system lays the foundation for the construction of the socioeconomic system.

**Figure 2:**
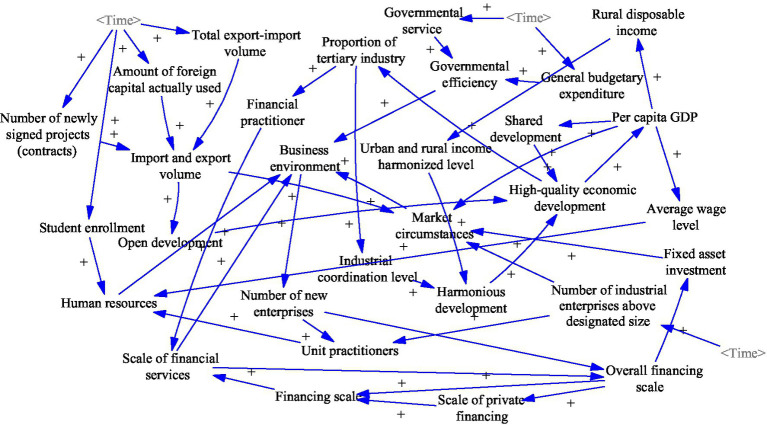
Economic development sub-system causality diagram.

The main causal feedback loops for the economic development sub-system are as follows. The specific causal relationships are shown in Figure 2.

1. Total export–import volume (+)/Number of newly signed projects (contracts) (+)/Amount of foreign capital used (+) → Import and export volume (+) → Open development (+) → High-quality economic development (+)
2. Total export–import volume (+)/Number of newly signed projects (contracts) (+)/Amount of foreign capital used (+) → Import and export volume (+) → Market circumstances (+) → Business environment (+)
3. Proportion of output value of tertiary industry (+) → Industrial coordination level (+) → Harmonious development (+) → High-quality economic development (+)
4. Proportion of output value of tertiary industry (+) → Financial practitioner (+) → Scale of financial services (+) → Overall financing scale (+) → Scale of private financing (+) → Financing scale (+) → Scale of financial services (+)
5. Business environment (+) → Number of new Enterprises (+) → Overall financing scale (+) → Fixed-asset investment (+) → Market circumstances (+) → Business environment (+)
6. Governmental service (+) → Governmental efficiency (+) → Business environment (+) → Number of new Enterprises (+) → Unit Practitioners (+) → Human resources (+) → Business environment (+)
7. High-quality economic development (+) → Per capita GDP (+) → Rural disposable income (+) → Urban and rural income harmonized level (+) → Harmonious development (+) → High-quality economic development (+).

### Public Service Sub-system Construction

The improvement of public services to achieve shared development is the goal and destination of economic development. It is also the starting and ending point of development. The improvement of the entrepreneurial environment is concerned with improving the total economic volume, the satisfaction with the quality of the economy, and the improvement in people’s living standards. The public services sub-system is studied in terms of electricity supply, education, and healthcare. It contains variables such as gas supply, electricity supply, and education expenditure. This sub-system is linked to the economic development sub-system and the resources and environment sub-system. The construction of this sub-system lays the foundation for the construction of the socioeconomic system.

**Figure 3:**
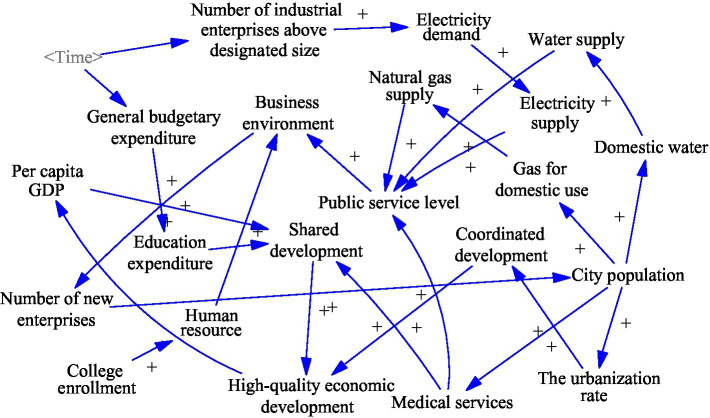
Public development sub-system causality diagram.

The main causal feedback loops in the public development sub-system are as follows. Figure 3 shows the specific causal relationships:

1. General budget expenditure (+) → Education spending (+) → Shared development (+) → High-quality economic development (+)
2. City population (+) → The urbanization rate (+) → Coordinated development (+) → High-quality economic development (+)
3. City population (+) → Medical Services (+) → Public service level (+) → Business environment (+)
4. Number of industrial enterprises above designated size (+) → Electricity demand (+) → Electricity supply (+) → Public service level (+) → Business environment (+)
5. Business environment (+) → Number of new Enterprises (+) → City population (+) → Domestic water (+) → Water supply (+) → Public service level (+) → Business environment (+)
6. City population (+) → Gas for domestic use (+) → Natural gas supply (+) → Public service level (+) → Business environment (+).

### Resource Environment Sub-system Construction

The improvement in the entrepreneurial environment is driving high-quality economic development, while resource consumption and environmental maintenance are receiving increasing attention. Green development emphasizes the integration of the economy with the environment and society and is a crucial way to achieve sustainable development. Therefore, resources and the environment are endogenous and constraining economic development factors. The resource and environment sub-system is studied in resource consumption, waste emissions, science and technology, and innovation. This sub-system is linked to the economic development sub-system, the public services sub-system, and the science and technology development sub-system. The construction of this sub-system lays the foundation for the construction of the socioeconomic system.

**Figure 4:**
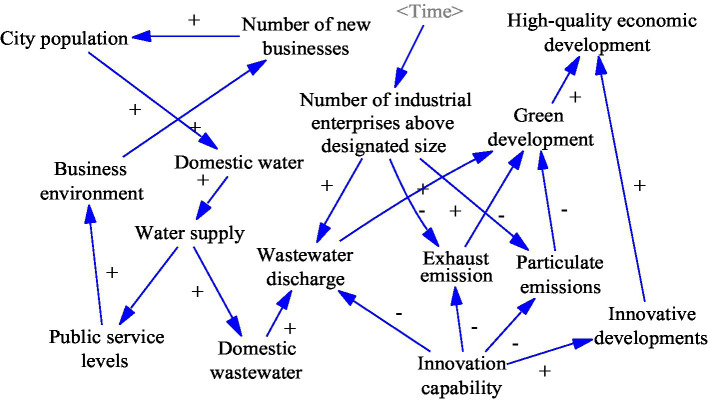
Resource environment sub-system causality diagram.

The main causal feedback loop of the resource and environment sub-system is as follows. Figure 4 shows the specific causal relationship.

1. Number of industrial enterprises above designated size (+) → Particulate emissions (+)/Wastewater discharge (+)/Exhaust emission (+) → Green development (−) → High-quality economic development (−)
2. Innovative capability (+) → Particulate emissions (−)/Wastewater discharge (−)/Exhaust emission (−) → Green development (+) → High-quality economic development (+)
3. Business environment (+) → Number of new Enterprises (+) → City population (+) → Domestic water (+) → Water supply (+) → Domestic wastewater (+) → Wastewater discharge (+) → Green development (−) → High-quality economic development (−).

### General Socioeconomic System Construction

**Figure 5:**
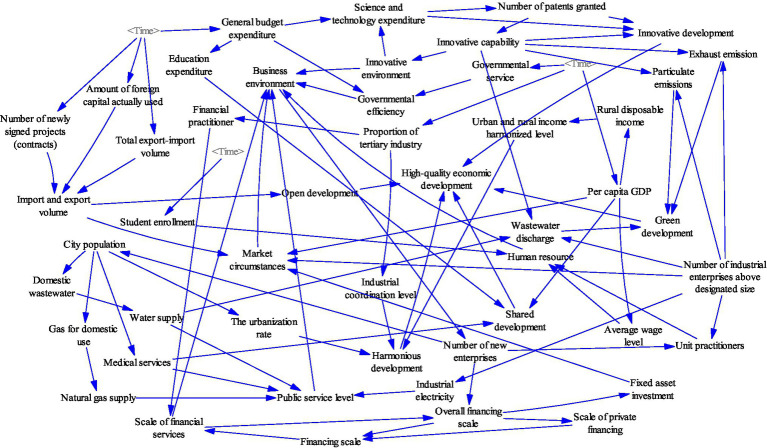
Total system causality diagram.

Based on the sub-systems constructed above, the article constructs a causal diagram of the whole system to reflect the relationships between the above sub-systems in the socioeconomic system, as Figure 5 shows. The establishment of cause-and-effect diagrams lays the foundation for the implementation of the simulation of system dynamics.

**Figure 6:**
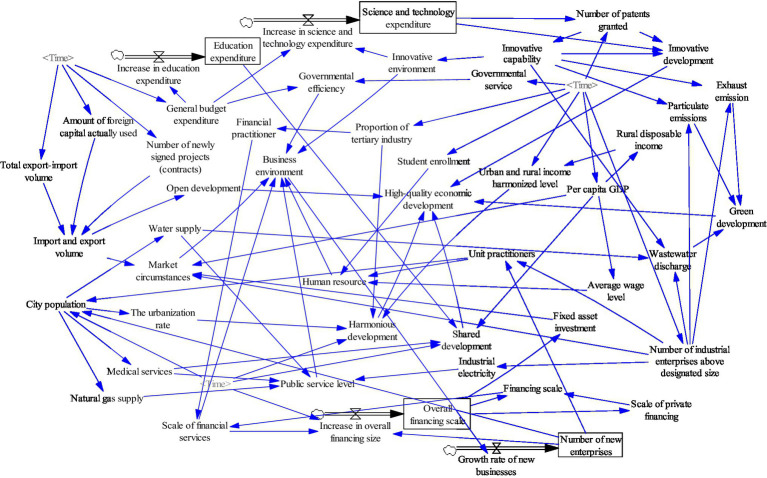
General system structure flow diagram.

The creation of structural flow diagrams is the most critical step in the simulation of system dynamics. The study introduces state variables, rate variables, and auxiliary variables to construct structural flow diagrams to clarify the nature of the variables based on causality diagrams and provide further clarity on the relationships between variables within the system (as shown in Figure 6). There are 50 variables in the model. There are four state variables, including education expenditure, science and technology expenditure, overall financing size, and the growth rate of the number of new firms. There are four rate variables: increase in science expenditure, increase in education expenditure, increase in overall financing size, and growth rate of enterprises. There are 42 auxiliary variables, including urbanization rate and average wage level.

## Results

### Quality Economic Development Assessment Results

Due to the potential correlation between indicators and the disparity in data across cities, the CRITIC method was chosen to assign objective weights to the indicators (see Supplementary Table 7 for details of the indicator assignments). Based on the CRITIC method, the weights of the above indicators were calculated to evaluate the quality of economic development of each city. Table 3 shows the results. Due to the large amount of data, only the top 20 cities have been selected for display each year.

**Table 3:** Results of the evaluation of high-quality economic development of cities.

Rank	2014		2015		2016		2017		2018	
1	Shenzhen	2.33	Shenzhen-	2.28	Shenzhen-	2.53	Shenzhen-	2.35	Shenzhen-	2.41
2	Zhuhai	1.40	Dongguan↑	1.54	Zhuhai↑	1.49	Zhuhai-	1.41	Zhuhai-	1.23
3	Dongguan	1.34	Zhuhai↓	1.50	Dongguan↓	1.45	Dongguan-	1.22	Dongguan-	1.20
4	Beijing	1.25	Beijing-	1.09	Tianjin↑	1.07	Beijing-	1.14	Zhongshan↑	0.98
5	Shanghai	1.09	Zhongshan↑	1.03	Zhongshan-	1.04	Shanghai↑	1.10	Beijing↓	0.95
6	Zhongshan	1.06	Shanghai↓	1.01	Shanghai-	1.01	Zhongshan↓	0.99	Shanghai↓	0.85
7	Tianjin	0.99	Tianjin-	0.94	Beijing↓	1.00	Zhoushan↑	0.74	Zhoushan-	0.77
8	Suzhou	0.85	Suzhou-	0.80	Changsha↑	0.79	Guangzhou↑	0.73	Guangzhou-	0.70
9	Zhoushan	0.84	Changsha↑	0.74	Suzhou↓	0.76	Changsha↓	0.73	Dalian↑	0.70
10	Changsha	0.75	Zhoushan↓	0.73	Zhoushan-	0.70	Hangzhou↑	0.67	Changsha↓	0.67
11	Wuhu	0.70	Hangzhou↑	0.71	Hangzhou-	0.69	Wuhu↑	0.67	Hangzhou↑	0.66
12	Ningbo	0.68	Wuhu↓	0.67	Guangzhou↑	0.68	Suzhou↓	0.64	Suzhou-	0.64
13	Dalian	0.67	Foshan↑	0.67	Wuhu↓	0.65	Wuhan↓	0.63	Wuhu↓	0.59
14	Foshan	0.61	Ningbo↓	0.64	Foshan↓	0.64	Foshan-	0.60	Wuhan↓	0.57
15	Hangzhou	0.61	Guangzhou↑	0.63	Ningbo↓	0.62	Dalian↑	0.60	Huizhou↑	0.57
16	Huizhou	0.60	Wuxi↑	0.56	Wuhan↑	0.60	Tianjin↑	0.59	Foshan↓	0.57
17	Guangzhou	0.59	Wuhan↑	0.53	Wuxi↓	0.51	Ningbo↓	0.55	Mudanjiang↑	0.52
18	Wuxi	0.57	Chengdu↑	0.51	Zhengzhou↑	0.50	Qingdao↑	0.53	Ningbo↓	0.51
19	Wuhan	0.48	Huizhou↓	0.47	Qingdao↑	0.48	Chengdu↑	0.51	Chengdu-	0.51
20	Weihai	0.48	Qingdao↑	0.44	Chengdu↓	0.43	Zhengzhou↓	0.47	Zhengzhou-	0.45

High-quality economic development measures a city’s economic development and makes a comprehensive judgment of economic development, urban resources and environment, and people’s lives. Table 3 shows that the top three cities from 2014 to 2018 were Shenzhen, Zhuhai, and Dongguan. Guangzhou has been moving upward among the top 20 cities, while Tianjin’s development has declined yearly. There is a difference in the number of top 20 cities in terms of the quality of economic development for those located in the south and in the north of China. Four of the top 20 cities in 2014 were located in northern China, and 16 of them were located in southern China. In 2018, the top 20 cities also included four cities located in northern China and 16 cities located in southern China. These figures indicate a regional imbalance in quality economic development. Cities in the south experienced stronger quality economic development than those in the north. The number of cities in the top 20 in terms of quality economic development varies by city group.

The top 20 cities in 2014 included seven cities in the Guangdong-Hong Kong-Macao Greater Bay Area, seven cities in the Yangtze River Delta city group, two cities in the Beijing-Tianjin-Hebei city group, two cities in the middle reaches of the Yangtze River city group, one city in the Central-Southern Area of Liaoning city group, one city in the Shandong Peninsula city group, and no cities in the Ha-Chang, Central Plains, or Chengdu-Chongqing city groups. The top 20 cities in 2018 included seven cities in the Guangdong-Hong Kong-Macao Greater Bay Area, six cities in the Yangtze River Delta city cluster, two cities in the Middle Yangtze River city cluster, one city in the Chengdu-Chongqing city cluster, one city in the Beijing-Tianjin-Hebei city cluster, one city in the Ha-Chang city cluster, one city in the Central-Southern Area of Liaoning city cluster, one city in the Central Plains city cluster, and no cities in the Shandong Peninsula city cluster. The status of high-quality economic development varies between city groups, with Guangdong-Hong Kong-Macao, and the Yangtze River Delta city groups doing relatively well.

As Table 4 shows, there are differences in economic development between urban agglomerations. Guangdong-Hong Kong-Macao and the Yangtze River Delta urban agglomerations are stable and well developed (in line with the results of the city measures above). Other city clusters show fluctuations of varying magnitudes, such as the Central-Southern Area of Liaoning city cluster, which fell from third place in 2014 to ninth place in 2018, and the Ha-Chang city cluster, which improved from fifth place in 2014 to third place in 2018. In addition, there are differences between cities within the same urban agglomeration. As Table 4 shows, Beijing has been ranked among the top cities for high-quality economic development. However, the Beijing-Tianjin-Hebei urban agglomeration has not been ranked well, being placed in the middle to bottom bracket in 2014 and 2015. Therefore, to improve the quality of economic development in urban agglomerations, there should be synergy and progress within the urban agglomerations. Cities that are better developed should take advantage of their successes to actively assist less well-developed cities.

**Table 4:** High-quality economic development in urban agglomerations.

City Cluster	2014		2015		2016		2017		2018	
	Value	Rank	Value	Rank	Value	Rank	Value	Rank	Value	Rank
Guangdong-Hong Kong-Macao	0.86	1	0.90	1	0.88	1	0.79	1	0.72	1
Yangtze River Delta	0.30	2	0.31	2	0.27	2	0.23	2	0.20	2
Central-Southern Liaoning	0.10	3	−0.01	3	−0.10	7	−0.15	7	−0.22	9
Shandong Peninsula	0.05	4	−0.02	4	−0.07	5	−0.08	6	−0.12	6
Harbin-Changchun	−0.06	5	−0.08	5	−0.05	4	−0.06	4	0.04	3
Beijing-Tianjin-Hebei	−0.07	6	−0.13	7	−0.03	3	−0.04	3	0.04	4
Middle Yangtze	−0.11	7	−0.11	6	−0.08	6	−0.06	5	−0.07	5
Chengdu and Chongqing	−0.20	8	−0.23	8	−0.26	9	−0.22	9	−0.17	8
Central Plains	−0.36	9	−0.28	9	−0.25	8	−0.19	8	−0.17	7

### Business Environment Assessment Results

The business environment of the cities included in the city cluster was evaluated through the CRITIC weighting method (see Supplementary Table 8 for details of the indicator weighting). Table 5 shows the results.

**Table 5:** City business environment evaluation results.

Ranking	2014		2015		2016		2017		2018	
1	Shanghai	4.16	Shanghai-	4.06	Shanghai-	3.69	Beijing↑	3.87	Beijing-	3.64
2	Beijing	3.86	Beijing-	3.72	Beijing-	3.62	Shanghai↓	3.79	Shanghai-	3.57
3	Shenzhen	2.40	Shenzhen-	2.57	Shenzhen-	2.80	Shenzhen-	3.00	Shenzhen-	2.99
4	Tianjin	2.07	Tianjin-	2.04	Tianjin-	2.19	Guangzhou↑	2.18	Guangzhou-	1.99
5	Guangzhou	1.95	Guangzhou-	1.99	Guangzhou-	2.12	Suzhou↑	1.81	Suzhou-	1.68
6	Suzhou	1.83	Chongqing↑	1.69	Chongqing-	1.57	Tianjin↓	1.77	Chongqing↑	1.67
7	Chongqing	1.76	Suzhou↓	1.65	Suzhou-	1.53	Chongqing↓	1.72	Chengdu↑	1.54
8	Hangzhou	1.44	Hangzhou-	1.48	Wuhan↑	1.51	Chengdu↑	1.62	Tianjin↓	1.52
9	Nanjing	1.36	Wuhan↑	1.26	Hangzhou↓	1.44	Hangzhou-	1.53	Wuhan↑	1.47
10	Wuhan	1.33	Nanjing↓	1.24	Chengdu↑	1.35	Wuhan↓	1.46	Hangzhou↓	1.45
11	Chengdu	1.21	Chengdu-	1.18	Nanjing↓	1.23	Nanjing-	1.22	Nanjing-	1.30
12	Ningbo	1.02	Dongguan↑	1.02	Dongguan-	1.04	Dongguan-	1.13	Ningbo↑	1.01
13	Dalian	1.02	Ningbo↓	0.98	Ningbo-	0.98	Zhengzhou↑	1.08	Zhengzhou-	1.00
14	Dongguan	0.99	Shenyang↑	0.89	Foshan↑	0.87	Changsha↑	1.06	Changsha-	0.95
15	Wuxi	0.92	Changsha↑	0.87	Changsha-	0.86	Ningbo↓	1.05	Wuxi↑	0.90
16	Shenyang	0.89	Dalian↓	0.86	Zhengzhou↑	0.81	Wuxi↑	0.89	Dongguan↓	0.90
17	Changsha	0.84	Wuxi↓	0.83	Wuxi-	0.81	Qingdao↑	0.83	Qingdao-	0.79
18	Qingdao	0.81	Foshan↑	0.83	Qingdao↑	0.76	Foshan↓	0.74	Jinan↑	0.65
19	Foshan	0.80	Qingdao↓	0.79	Jinan↑	0.58	Hefei↑	0.70	Hefei-	0.62
20	Zhengzhou	0.74	Zhengzhou-	0.79	Changzhou↑	0.54	Jinan↓	0.60	Foshan↓	0.53

The business environment is a comprehensive evaluation of regional government efficiency, human resources, financial services, public services, market, and innovation. As Table 5 shows, the top three cities from 2014 to 2018 were Beijing, Shanghai, and Shenzhen. From 2014 to 2016, Shanghai was the top city, and Beijing was in second place. From 2017 to 2018, Beijing overtook Shanghai to be first. Shenzhen has been in the third position. Unlike the situation with high-quality economic development, more provincial capitals and municipalities directly under the central government are ranked at the top of the list for the business environment. This indicates that the central location of provincial capitals and municipalities directly under the central government impacts the development of the business environment of urban agglomerations.

There is a difference in the number of top 20 cities in terms of business environment located in southern and northern China. The top 20 cities in 2014 included six cities in northern China and 14 cities in southern China. Among the top 20 cities in 2018, there were five cities in northern China and 15 cities in southern China. It is clear that there is a geographical imbalance in the business environment, with southern Chinese cities having a better business environment than northern Chinese cities. The number of cities in the top 20 cities for business environment development varies by city group.

As Table 6 shows, there are differences in the development of the business environment between urban agglomerations. Guangdong-Hong Kong-Macau, and the Yangtze River Delta city cluster have consistently ranked first and second in the business environment. The business environment in the Greater Bay Area of Guangdong-Hong Kong-Macau is better than in the Yangtze River Delta city cluster. In terms of the number of top 20 business environment rankings, there are more Yangtze River Delta city clusters than in the Guangdong-Hong Kong-Macau Greater Bay Area. Therefore, each city cluster should focus on the synergistic development among cities within the cluster to achieve an overall improvement in the business environment of the city cluster.

**Table 6:** Business environment in urban agglomerations.

City Clusters	2014		2015		2016		2017		2018	
	Value	Rank	Value	Rank	Value	Rank	Value	Rank	Value	Rank
Guangdong-Hong Kong-Macao	0.73	1	0.90	1	0.88	1	0.79	1	0.72	1
Yangtze River Delta	0.45	2	0.31	2	0.27	2	0.23	2	0.20	2
Central-southern Liaoning	0.43	3	−0.13	7	−0.03	3	−0.04	3	0.04	4
Shandong Peninsula	0.03	5	−0.01	3	−0.10	7	−0.15	7	−0.22	9
Harbin-Changchun	0.16	4	−0.02	4	−0.07	5	−0.08	6	−0.12	6
Beijing-Tianjin-Hebei	−0.22	6	−0.08	5	−0.05	4	−0.06	4	0.04	3
Middle Yangtze	−0.27	8	−0.11	6	−0.08	6	−0.06	5	−0.07	5
Chengdu and Chongqing	−0.27	7	−0.23	8	−0.26	9	−0.22	9	−0.17	8
Central Plains	−0.41	9	−0.28	9	−0.25	8	−0.19	8	−0.17	7

The Beijing-Tianjin-Hebei, Shandong Peninsula, Ha-Chang, and Middle Yangtze River city groups ranked medium. South-Central Liaoning, Chengdu-Chongqing, and Central Plains urban agglomerations ranked low. The magnitude of fluctuations varies from city cluster to city cluster. For example, the Central-Southern Area of Liaoning city cluster fluctuated from fifth place in 2014 to ninth place in 2018, and the Ha-Chang city cluster improved from sixth place in 2014 to third place in 2018. A combination of the results of the evaluation of the business environment of individual cities and city clusters shows that there are differences in the business environments of cities within city clusters. The central cities in the cluster (Shanghai, Beijing, Shenzhen, Guangzhou, Chongqing, Hangzhou, Wuhan, etc.) have a better business environment. For example, Chengdu and Chongqing are consistently ranked among the top cities studied, but the ranking of the Chengdu-Chongqing cluster is impressive. Therefore, to improve the business environment of city clusters, there should be synergistic development within the cluster. The central city should drive the development of the business environment of the surrounding cities.

### Simulation Prediction and Sensitivity Analysis Based on System Dynamics

This paper introduces system dynamics to simulate the socioeconomic system to observe the changes in the number of new enterprises and the quality development of the economy (Ding and Ye, 2017). Using city cluster data from 2014 to 2018, the paper simulates the business environment of city clusters and the economic quality development in 2019–2021. The simulation equation for each urban agglomeration differs according to different regions’ resource endowments, economic structures, development goals, and development approaches. Therefore, the economic and social development of each of the nine urban agglomerations is modeled separately by drawing a simulation network of each one. The nine urban agglomerations’ regional entrepreneurship and economic quality development changes are compared and analyzed. This research further explores the sensitive factors affecting the business environment and economic quality development in different urban agglomerations to reveal the characteristics and ways of influencing regional entrepreneurship and economic quality changes when a particular factor of the business environment is in the regulatory system.

Before conducting the simulation prediction and sensitivity analysis, the system dynamics model needs to be tested by historical simulation. The results are shown in Supplementary Tables 9 and 10. When the simulated values of the business environment and economic quality development from 2014 to 2018 are compared with the historical values, it can be seen that the error between the simulated values and the historical values is small, within 5% in all years. The model passed the validity test.

Due to space limitations, only three cases were set up in this paper. The three scenarios regulate the indicators of the business environment, financial services scale, and innovation environment, respectively. The scenarios and simulation results are shown below.

#### Scenario a

Sensitivity analysis of entrepreneurship and high-quality economic development in urban agglomerations by increasing business environment by 10% from the original data for urban agglomerations, with all other variables held constant.

#### Scenario b

Sensitivity analysis of entrepreneurship and high-quality economic development in urban agglomerations by increasing the size of financial services by 10% from the original data for urban agglomerations, with all other variables held constant.

#### Scenario c

Sensitivity analysis of entrepreneurship and high-quality economic development in urban agglomerations by raising the innovation environment by 10% from the benchmark data for urban agglomerations, with all other variables held constant.

#### Simulation Forecasting and Sensitivity Analysis of Entrepreneurship in Urban Agglomerations

This paper uses the number of new enterprises per year to measure entrepreneurship in the region. All other factors being equal, the direction and magnitude of change in new business creation vary across city clusters when the business environment or a factor within it is increased by 10%. For “Scenario a” (changes in the business environment), there was no significant change in the results of the enhanced business environment in the Guangdong-Hong Kong-Macao Greater Bay Area and Shandong Peninsula city cluster. This shows that the entrepreneurial situation in the above two urban agglomerations is not sensitive to this factor. The Beijing-Tianjin-Hebei, Yangtze River Delta, Central-Southern Area of Liaoning, Ha-Chang, and Chengdu-Chongqing city clusters decreased the number of new enterprises each year following their improved business environment. This may be because improving the entrepreneurial situation in these areas requires a combination of factors that cannot be met by regulating the business environment alone. The number of new businesses per year in the middle reaches of the Yangtze River and the Central Plains urban agglomeration rose significantly since the upgrading of the business environment, indicating that entrepreneurship in the region has improved significantly since the upgrading of the business environment.

In “Scenario b”, all urban agglomerations are sensitive to this element, except for the Shandong Peninsula, which shows no significant change in terms of the scale of financial services. Among them, after a 10% increase in the scale of regional financial services in Beijing-Tianjin-Hebei, Guangdong-Hong Kong-Macao, the middle reaches of the Yangtze River, and the Chengdu-Chongqing urban agglomeration, the entrepreneurial situation was initially insignificant, then recovered a few years later. After a 10% increase in the size of regional financial services in the Yangtze River Delta, South-Central Liaoning, Ha-Chang, and Central Plains urban agglomerations, the entrepreneurial situation showed a decrease.

Regarding changes in the innovation environment in “Scenario c”, all urban agglomerations experienced some degree of change in entrepreneurship, except for the Shandong Peninsula and the Middle Yangtze River urban agglomerations, which were less sensitive to changes in the innovation environment. Among them, Guangdong-Hong Kong-Macao, the Yangtze River Delta, and the Central Plains urban agglomeration saw a boost in entrepreneurship following improvements in the innovation environment. The innovation environment in the Beijing-Tianjin-Hebei, Central-Southern Area of Liaoning, Ha-Chang, and Chengdu-Chongqing city clusters declined following improvements in entrepreneurship. The main reason for this is that a single improved innovation environment will not meet the needs of the improved entrepreneurial situation. In other words, the city’s mass innovation environment is not the only factor needed to enhance regional entrepreneurship.

**Figure 7:**
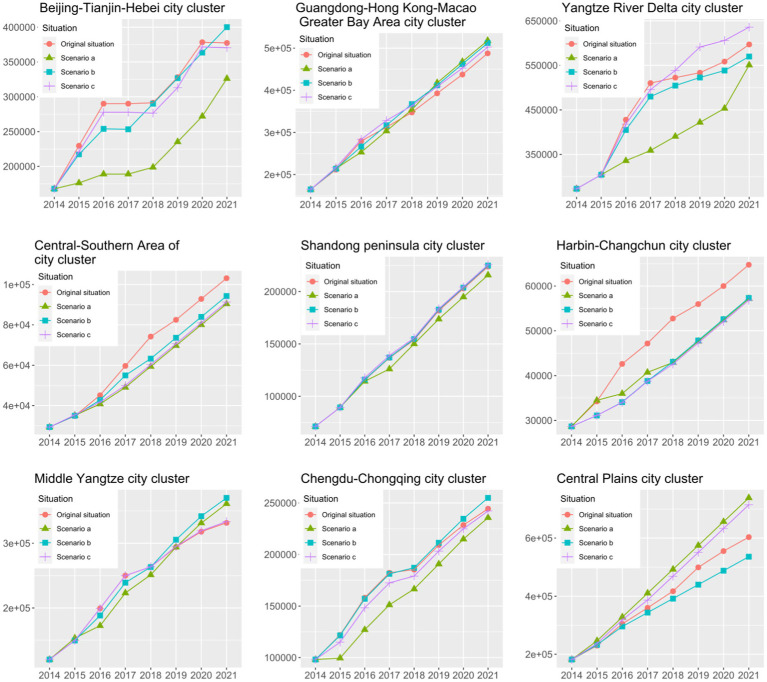
Results of simulation projections and sensitivity analysis of the number of new enterprises per year in urban agglomerations.

For each urban agglomeration, the sensitivity of entrepreneurship to various factors varies within different urban agglomerations, as illustrated by the Yangtze River Delta urban agglomeration. As Figure 7 shows, “Scenario a” (improved business environment) leads to significantly more changes in entrepreneurship than the other scenarios. The Yangtze River Delta city cluster is more sensitive to overall improvements in the business environment than to the scale of financial services and innovation. Therefore, when it comes to improving the entrepreneurial situation in the Yangtze River Delta, priority should be given to improving the overall business environment rather than the scale of financial services and the innovation environment.

#### Simulation Forecasting and Sensitivity Analysis for High-Quality Economic Development of Urban Agglomerations

In terms of high-quality economic development, the direction and magnitude of change in the indicators of high-quality economic development vary across different urban agglomerations when a particular factor increases by 10%, all other factors being equal. In “Scenario a”, the changes in economic quality development following changes in the business environment in the Guangdong-Hong Kong-Macao Bay Area, the middle reaches of the Yangtze River, and the Chengdu-Chongqing urban agglomerations are insignificant. In other words, these three city clusters are not sensitive to fluctuations in economic quality development after changes in the business environment. Other urban agglomerations experienced a degree of fluctuation in quality economic development following changes in the business environment. Among them, the changes in the business environment in the Beijing-Tianjin-Hebei, Central-Southern Area of Liaoning, and Ha-Chang city clusters promote high-quality economic development in the short term, while the long-term promotion effect disappears. Changes in the business environment in other urban agglomerations inhibit quality economic development. The reasons for this may be, for example, that the entry of new businesses increases environmental pressures in urban agglomerations.

Regarding the scale of financial services in “Scenario b”, changes in the scale of financial services in the urban agglomerations of Beijing-Tianjin-Hebei, Central-South Liaoning, and Central Plains promote high-quality economic development in the region. The Yangtze River Delta and Ha-Chang urban agglomerations have disimproved in terms of quality economic development following changes in the scale of financial services. The high-quality economic development of the above urban agglomerations is sensitive to changes in the scale of financial services. Changes in the scale of financial services in Guangdong-Hong Kong-Macao, the Shandong Peninsula, the middle reaches of the Yangtze River, and the Chengdu-Chongqing urban agglomerations are insignificant in terms of changes in the quality of economic development. In other words, these four urban agglomerations are not sensitive to changes in the scale of financial services affecting changes in the quality of economic development.

Regarding changes in the innovation environment in “Scenario c”, the Beijing-Tianjin-Hebei, Yangtze River Delta, Central-Southern Area of Liaoning, and Shandong Peninsula city clusters promoted high-quality economic development following changes in the innovation environment. Economic quality development deteriorated following changes in the innovation environment in the Ha-Chang, Chengdu-Chongqing, and Central Plains city clusters. The state of high-quality economic development in the above urban agglomerations is sensitive to changes in the innovation environment. Changes in the innovation environment in Guangdong-Hong Kong-Macao and the Yangtze River’s middle reaches are not significant in terms of changes in economic quality development. These four city clusters are not sensitive to changes in the innovation environment in relation to changes in high-quality economic development. Ding and Ye (2017) also illustrate regional heterogeneity in the relationship between innovation and high-quality economic development based on interprovincial data in China.

**Figure 8:**
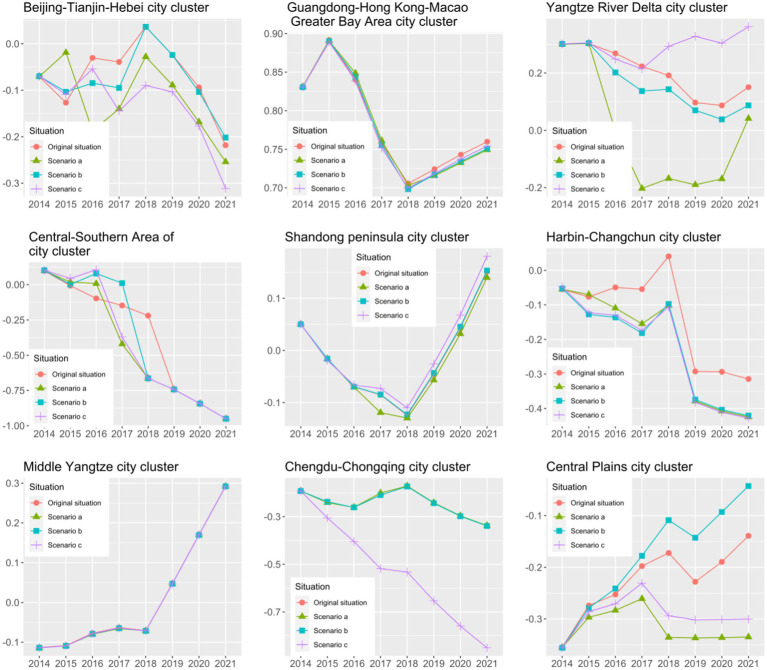
Results of high-quality economic development simulation forecasts and sensitivity analysis for urban agglomerations.

For each urban agglomeration, the sensitivity of high-quality economic development within different urban agglomerations to various factors varies, as illustrated by the Chengdu-Chongqing urban agglomeration. As Figure 8 shows, “Scenario c” (changes in the innovation environment) leads to significantly more changes in economic quality development than the other scenarios affecting economic quality development in this urban agglomeration. The intensity of sensitivity to the innovation environment in the Chengdu-Chongqing urban agglomeration is higher than the sensitivity to the business environment and the scale of financial services. Therefore, if we want to improve the quality of economic development in the Chengdu-Chongqing urban agglomeration, we should prioritize changes in the innovation environment rather than the scale of the business environment and financial services.

### Further Analysis

The combination of changes in the business environment, regional entrepreneurship, and quality economic development shows that while the scale of financial services is an indicator of the business environment, it can also lead to changes in quality economic development. From a system perspective, each indicator is a component of the system, and changes in the system are linked and dynamic. Thus, the scale of financial services will affect changes in the business environment and lead to changes in the quality of regional entrepreneurship and economic development. For the same reason, changes in the innovation environment also impact the business environment, regional entrepreneurship, and quality economic development. In comparison to the three scenarios, the sensitivity of regional entrepreneurship to quality economic development differs depending on the resource endowment, economic structure, development goals, and development approach of the urban agglomerations in which they are located. The study could not make a macro-level case for the clear-cut conclusion that the impact on factors in one of the scenarios was more vital than in the remaining two. It is valid to apply the network model to estimate the economic development opportunities in a region (Antipov et al., 2019). Therefore, more attention should be paid to micro-city adaptation when formulating corresponding policy measures to improve the business environment and enhance quality economic development. Policy measures adapted to the sensitive characteristics of each region should be tailored to local conditions.

The relationship between the business environment, the scale of financial services, innovation, regional entrepreneurship, and high-quality economic development is complex. In theory, the business environment, the scale of financial services, and the improvement of the entrepreneurial environment will improve the regional entrepreneurial situation and enhance economic development (Radović-Marković et al., 2019; Jang et al., 2020; Pradhan et al., 2020; Srivastava et al., 2021). However, the study found that due to regional heterogeneity, not all of the above three factors have a positive effect on regional entrepreneurship and quality economic development. The same conclusion has been reached by other scholars (Ding and Ye, 2017; Zhou et al., 2021).

Based on the above empirical results, the changes in entrepreneurship and quality economic development are asymmetrical, where the changes in the three scenarios for a given urban agglomeration. Changes in one factor in some urban agglomerations negatively affect entrepreneurship and quality economic development. For example, the Central Plains urban agglomeration in “Scenario b” (increasing the scale of financial services) leads to lower regional entrepreneurship but promotes high-quality economic development. The impact of a change in a factor in some urban agglomerations on entrepreneurship is congruent with quality economic development. For example, the Yangtze River Delta city cluster in “Scenario c” state (enhancing the innovation environment) enables an enhanced entrepreneurial environment while promoting high-quality economic development.

The south-central Liaoning and Ha-Chang urban agglomerations are located in the Northeast of China. As Figures 7, 8 show, the south-central Liaoning and Ha-Chang urban agglomerations do not show significant growth under the three simulation scenarios. The possible reason is that the above three scenarios are not the factors driving the economic development of Northeast China. As an old industrial base, the Northeast is facing serious population aging, low birth rate, and migration. Ren et al. (2020) found that human resources are a key factor in improving economic development in Northeast China.

In summary, the variation in both entrepreneurship and quality economic development is determined by the variation in the internal factors of the system. The relationship and variation between the two are dynamic, complex, and multifactorial and cannot be summarized in simple linear or other single models. Pidorycheva (2020) also proposed an innovation ecosystem for a region (economic area) and developed a conceptual model of a regional innovation ecosystem from a systems theory perspective. It is possible to demonstrate whether a factor is sensitive for urban agglomerations. However, the sensitivity analysis of other indicators and how to quickly identify sensitive factors affecting the research subjects to enhance regional entrepreneurship and improve the quality of economic development are limitations of this paper. They are worthy of continued exploration in the future.

## Discussion and Conclusion

This study selected raw data from 146 Chinese cities, at the prefecture level and above, containing seven national city clusters and two local city clusters from 2014 to 2018. It constructed a city-level business environment and economic quality development indicator system, respectively, and a city-level socioeconomic system in China. This study designed causality diagrams for each sub-system in the socioeconomic system and the total system, summarizing the structural flow diagram of the total system. The study applied the CRITIC empowerment method to evaluate the business environment and quality economic development of the selected cities and city clusters. A sensitivity analysis based on system dynamics was conducted on regional entrepreneurship and quality economic development to explore the trends in entrepreneurship and quality economic development when the business environment, the scale of financial services, and the innovation environment change.

This paper innovatively applies system dynamics to the issues of the business environment, regional entrepreneurship, and high-quality economic development. It proposes a city-level evaluation system for high-quality economic development. The discussion and conclusions of the current study are as follows.

The evaluation of the business environment and quality economic development found that the Guangdong-Hong Kong-Macao Bay Area and the Yangtze River Delta city cluster are successful in terms of the business environment and quality economic development. The business environment and the quality of economic development in other city groups changed over the years. There are differences between cities in their city clusters, with the central cities having a better business environment and high-quality economic development. In a competitive market environment, entrepreneurs are very concerned about where to set up their businesses (McMullen et al., 2016). Therefore, to improve the business environment and the quality of economic development in urban agglomerations, it is necessary to develop synergies and progress simultaneously. Li and Phelps (2019) explored the value and positive role of Shanghai in the Yangtze River Delta city cluster from the perspective of economic geography. Cities in a better state of development should take advantage of their progress to actively assist cities in a less developed state (Bondarenko et al., 2019).

The essence of the business environment’s involvement in promoting entrepreneurial situations and high-quality economic development is to optimize the complex socioeconomic system by promoting coordination and progress among the sub-systems, thereby achieving a comprehensive improvement of the total system efficiency. The socioeconomic system is a multi-level, multi-variable, and multi-subject complex system in which a change in one of the factors will produce linked, dynamic changes in the system. Many studies have demonstrated that the application of system dynamics can explore macroeconomic, corporate business, and other multi-level policy structure issues (Forrester et al., 1976; Qi and Chang, 2011; Marzouk and Azab, 2014). A sensitivity analysis based on a system dynamics model shows that changes in regional entrepreneurship following a change in a factor in the system are asymmetrical in relation to changes in high-quality economic development, with changes in both depending on changes in factors within the system.

This study explores entrepreneurship and economic quality development in urban clusters when the business environment, the scale of financial services, and the innovation environment change, respectively. The sensitivities of regional entrepreneurship and quality economic development and how they are affected by the changes vary according to the resource endowments, economic structure, development goals, and development approaches of the urban agglomerations in which they are located. Due to the different resource endowment, economic infrastructure, and technology base, differentiated technology strategies should be implemented by region (Zhou et al., 2021). When formulating policy measures from a macro perspective to improve the business environment accordingly and to enhance the entrepreneurial situation and quality economic development of a region, more attention should be paid to the adaptability of micro-cities, adapting policy measures to the local conditions and the sensitivity characteristics of each region.

## Data Availability Statement

The original contributions presented in the study are included in the article/Supplementary Material, further inquiries can be directed to the corresponding author.

## Author Contributions

CG and XL contributed the system dynamics simulation part of the article. CL and QX contributed the evaluation and analysis of the business environment and high-quality economic development. All authors contributed to this article and approved the submitted version.

## Funding

The Open Access Publication Fees are funded by the National Natural Science Foundation of China (62073007) and Beijing Social Science Fund (SZ202210005004).

## Conflict of Interest

The authors declare that the research was conducted in the absence of any commercial or financial relationships that could be construed as a potential conflict of interest.

## Publisher’s Note

All claims expressed in this article are solely those of the authors and do not necessarily represent those of their affiliated organizations, or those of the publisher, the editors and the reviewers. Any product that may be evaluated in this article, or claim that may be made by its manufacturer, is not guaranteed or endorsed by the publisher.

## Supplementary Material

The Supplementary Material for this article can be found online at: https://www.frontiersin.org/articles/10.3389/fpsyg.2022.905590/full#supplementary-material
