# Supplementary material for: Regional Entrepreneurship, Business Environment, and High-Quality Economic Development: An Empirical Analysis of Nine Urban Agglomerations in China
Source: Front Psychol. 2022 May 17;13:905590. doi: 10.3389/fpsyg.2022.905590 (PMC9152422; doi:10.3389/fpsyg.2022.905590)
Supplement: Supplementary file 1 [file Data_Sheet_1.docx]

Supplementary Material

# Evaluation of Business Environment and Economic Quality Development Based on the CRITIC Method

In this study, the CRITIC weighting method was used to define the amount of information contained in an indicator by calculating variability and conflicting indicators. The indicators are assigned weights based on the amount of information in the indicators relative to all the indicators in the sample. The specific calculation steps are as follows:

Positivization process:

$x'_{ij}=-\left|x_{ij}-\bar{x}\right|$ （1）

With *n* samples and *m* indicators, $x_{ij}$ is the *j*th indicator of sample *i* (*i* = 1,2,…,*n*; *j* = 1,2,…,*m*) and $\bar{x}$ is the mean of sample *i*.

Dimensionless processing:

$Z_{ij}\text{=}\frac{x_{ij}-\min\left\{x_{1j},,,,x_{nj}\right\}}{\max\left\{x_{1j},,,,x_{nj}\right\}-\min\left\{x_{1j},,,,x_{nj}\right\}}$ （2）

$z_{ij}$ is the value of the *j*th indicator of the *i*th sample (*i* = 1,2,…,*n*; *j* = 1,2,…,*m*) after dimensionless processing.

The variability of the indicator is calculated as:

$\left\{\begin{matrix} \bar{x}_{j}=\frac{1}{n}\sum\begin{matrix} n \\ i=1 \end{matrix}x_{ij} \\ S_{j}=\sqrt{\frac{\sum\begin{matrix} n \\ i=1 \end{matrix}(x_{ij}-\bar{x}_{j})^{2}}{n-1}} \end{matrix}\right.$ （3）

*S_j_* is the standard deviation of the *j*th indicator (*i* = 1,2,…,*n*; *j* = 1,2,…,*m*) of the *i*th sample. The CRITIC assignment method uses the standard deviation to measure the variation within an indicator and considers that the larger the standard deviation, the more information the indicator contains.

The conflicting nature of the indicators is calculated as:

$R_{j}\text{=}\sum\begin{matrix} p \\ i=1 \end{matrix}(1-r_{ij})$ （4）

*r_ij_* is the correlation coefficient between indicators *j* of sample *i*.

Indicator information calculation:

$C_{j}=S_{j}\sum\begin{matrix} p \\ i=1 \end{matrix}(1-r_{ij})=S_{j}\times R_{j}$ （5）

$C_{j}$is the amount of information contained in the *j*th indicator of sample *i* (*i* = 1,2,...,n; *j* = 1,2,...,m).

Indicator Empowerment:

$W_{j}=\frac{C_{j}}{\sum\begin{matrix} p \\ j=1 \end{matrix}C_{j}}$ （6）

$W_{j}$ is the weight of the *j*th indicator (*i* = 1,2,…,*n*; *j* = 1,2,…,*m*) of sample *i*.

# Business Environment and Economic Quality Development Indicators Assignment Results

The results of business environment and economic quality development indicators are assigned as follows:

Table 7 Calculation results of the weight of CRITIC empowerment method for high-quality economic development

|  | 2014 | 2015 | 2016 | 2017 | 2018 |
| --- | --- | --- | --- | --- | --- |
| Particulate emissions | 8.47% | 9.48% | 8.66% | 8.39% | 7.77% |
| Wastewater discharge | 9.12% | 9.06% | 9.30% | 9.42% | 8.76% |
| Exhaust emission | 7.78% | 8.04% | 7.82% | 7.54% | 7.30% |
| Innovation inputs | 7.41% | 6.74% | 6.81% | 6.71% | 6.52% |
| Innovative outputs | 5.96% | 5.86% | 5.76% | 6.00% | 6.19% |
| Level of industry coordination | 13.63% | 13.40% | 13.61% | 13.16% | 11.58% |
| The urbanization rate | 6.85% | 6.91% | 7.19% | 7.09% | 9.02% |
| Urban and rural income harmonized level | 7.16% | 7.32% | 7.29% | 7.49% | 7.20% |
| Import and export scale | 6.11% | 5.96% | 6.15% | 7.21% | 7.84% |
| Foreign trade dependence | 8.89% | 8.67% | 8.75% | 8.50% | 8.57% |
| GDP per capita | 6.32% | 6.28% | 6.20% | 6.17% | 6.58% |
| Educational situation | 5.76% | 5.65% | 5.66% | 5.74% | 5.97% |
| Medical services | 6.54% | 6.64% | 6.80% | 6.59% | 6.70% |

Table 8 Calculation results of CRITIC weighting method for city business environment

|  | 2014 | 2015 | 2016 | 2017 | 2018 |
| --- | --- | --- | --- | --- | --- |
| General budgetary expenditure | 3.69% | 3.61% | 3.65% | 3.72% | 3.69% |
| Governmental efficiency | 7.56% | 9.03% | 10.10% | 6.73% | 6.70% |
| Average wage level | 4.53% | 4.74% | 4.39% | 4.90% | 4.29% |
| Student enrollment | 6.25% | 6.21% | 6.08% | 6.19% | 5.88% |
| Unit practitioners | 4.04% | 4.15% | 3.54% | 3.90% | 3.52% |
| Financial practitioners | 4.19% | 4.15% | 4.16% | 4.42% | 4.15% |
| Scale of private financing | 3.49% | 3.62% | 3.62% | 3.58% | 3.64% |
| Overall financing scale | 3.13% | 3.04% | 3.05% | 3.08% | 3.11% |
| Natural gas supply | 4.70% | 4.68% | 4.74% | 4.69% | 4.63% |
| Water supply | 4.07% | 3.86% | 3.89% | 3.88% | 7.99% |
| Industrial electricity | 4.69% | 4.51% | 4.44% | 6.02% | 5.56% |
| Medical services | 6.93% | 6.83% | 6.64% | 6.62% | 6.26% |
| Per capita GDP | 6.68% | 6.26% | 5.97% | 6.18% | 5.62% |
| Fixed asset investment | 4.96% | 5.14% | 5.22% | 5.73% | 5.13% |
| Amount of foreign capital used | 4.61% | 4.10% | 4.87% | 3.79% | 3.89% |
| Number of new contracts signed | 5.21% | 5.36% | 5.31% | 6.06% | 6.65% |
| Number of industrial enterprises above designated size | 5.11% | 5.18% | 5.26% | 5.55% | 5.20% |
| Scientific expenditure | 5.73% | 5.35% | 5.36% | 5.13% | 4.76% |
| Number of patents granted | 3.86% | 3.67% | 3.40% | 3.33% | 3.48% |
| Innovation capability index | 6.56% | 6.50% | 6.31% | 6.51% | 5.86% |

# System Dynamics Simulation Data

Historical simulation tests of the system dynamics model are required before conducting simulation forecasting and sensitivity analysis. The business environment and economic quality development from 2014–2018 were used as the test variables. Tables 9 and 10 show the test results.

Table 9 Data table of historical test of business environment of urban clusters

|  |  | **2014** | **2015** | **2016** | **2017** | **2018** |
| --- | --- | --- | --- | --- | --- | --- |
| **Beijing-Tianjin-Hebei** | True Value | 0.431068 | 0.433025 | 0.571743 | 0.487369 | 0.406883 |
|  | Simulation value | 0.435848 | 0.437088 | 0.593511 | 0.485145 | 0.396916 |
|  | Relative Error | 1.11% | 0.94% | 3.81% | -0.46% | -2.45% |
| **Guangdong-Hong Kong-Macao** | True Value | 0.733035 | 0.784453 | 0.867989 | 0.839619 | 0.735518 |
|  | Simulation value | 0.736432 | 0.778521 | 0.848838 | 0.827154 | 0.726401 |
|  | Relative Error | 0.46% | -0.76% | -2.21% | -1.48% | -1.24% |
| **Yangtze River Delta** | True Value | 0.45405 | 0.439929 | 0.363173 | 0.426272 | 0.41788 |
|  | Simulation value | 0.46468 | 0.439606 | 0.372227 | 0.421897 | 0.422625 |
|  | Relative Error | 2.34% | -0.07% | 2.49% | -1.03% | 1.14% |
| **Central-southern Liaoning** | True Value | 0.029228 | 0.057171 | -0.25449 | -0.2527 | -0.2587 |
|  | Simulation value | 0.028199 | 0.057981 | -0.24649 | -0.24649 | -0.26986 |
|  | Relative Error | -3.52% | 1.42% | -3.14% | -2.46% | 4.31% |
| **Shandong Peninsula** | True Value | 0.163446 | 0.128948 | 0.113585 | 0.120203 | 0.133411 |
|  | Simulation value | 0.166156 | 0.127833 | 0.109049 | 0.116281 | 0.130922 |
|  | Relative Error | 1.66% | -0.86% | -3.99% | -3.26% | -1.87% |
| **Harbin-Changchun** | True Value | -0.2211 | -0.24041 | -0.34679 | -0.25797 | -0.29929 |
|  | Simulation value | -0.22505 | -0.23818 | -0.3418 | -0.2481 | -0.3065 |
|  | Relative Error | 1.78% | -0.93% | -1.45% | -3.82% | 2.41% |
| **Middle Yangtze** | True Value | -0.26873 | -0.26819 | -0.18031 | -0.2288 | -0.20682 |
|  | Simulation value | -0.2736 | -0.2606 | -0.1791 | -0.2278 | -0.20571 |
|  | Relative Error | 1.80% | -2.82% | -0.67% | -0.42% | -0.54% |
| **Chengdu-Chongqing** | True Value | -0.26835 | -0.33132 | -0.18592 | -0.23889 | -0.18978 |
|  | Simulation value | -0.2691 | -0.3266 | -0.1798 | -0.2296 | -0.1867 |
|  | Relative Error | 0.26% | -1.43% | -3.30% | -3.88% | -1.65% |
| **Central Plains** | True Value | -0.40874 | -0.36511 | -0.39589 | -0.37256 | -0.33914 |
|  | Simulation value | -0.4102 | -0.3667 | -0.3792 | -0.3709 | -0.3487 |
|  | Relative Error | 0.37% | 0.44% | -4.23% | -0.43% | 2.82% |

Table 10 Data table of historical test of economic quality development of urban agglomerations

|  |  | **2014** | **2015** | **2016** | **2017** | **2018** |
| --- | --- | --- | --- | --- | --- | --- |
| **Beijing-Tianjin-Hebei** | True Value | -0.06941 | -0.13016 | -0.03175 | -0.03786 | 0.036907 |
|  | Simulation value | -0.070567 | -0.126681 | -0.030595 | -0.039281 | 0.0367494 |
|  | Relative Error | 1.67% | -2.67% | -3.63% | 3.74% | -0.43% |
| **Guangdong-Hong Kong-Macao** | True Value | 0.864195 | 0.899004 | 0.878923 | 0.793447 | 0.723277 |
|  | Simulation value | 0.831032 | 0.891523 | 0.840139 | 0.757815 | 0.705521 |
|  | Relative Error | -3.84% | -0.83% | -4.41% | -4.49% | -2.45% |
| **Yangtze River Delta** | True Value | 0.299679 | 0.30581 | 0.274189 | 0.233301 | 0.198506 |
|  | Simulation value | 0.300613 | 0.303296 | 0.268447 | 0.223115 | 0.191549 |
|  | Relative Error | 0.31% | -0.82% | -2.09% | -4.37% | -3.50% |
| **Central-southern Liaoning** | True Value | 0.100331 | -0.00719 | -0.09768 | -0.15249 | -0.21568 |
|  | Simulation value | 0.099118 | -0.00717 | -0.09754 | -0.14749 | -0.21957 |
|  | Relative Error | -1.21% | -0.35% | -0.14% | -3.28% | 1.80% |
| **Shandong Peninsula** | True Value | 0.051324 | -0.01617 | -0.06755 | -0.08046 | -0.12468 |
|  | Simulation value | 0.050178 | -0.01604 | -0.06983 | -0.08324 | -0.12442 |
|  | Relative Error | -2.23% | -0.81% | 3.37% | 3.45% | -0.21% |
| **Harbin-Changchun** | True Value | -0.05857 | -0.076 | -0.04911 | -0.0567 | 0.039914 |
|  | Simulation value | -0.0552 | -0.07686 | -0.0495 | -0.0547 | 0.0407 |
|  | Relative Error | -4.57% | 1.12% | 0.90% | -3.58% | 1.96% |
| **Middle Yangtze** | True Value | -0.11459 | -0.10652 | -0.07901 | -0.06034 | -0.07266 |
|  | Simulation value | -0.1135 | -0.1086 | -0.0775 | -0.06225 | -0.07083 |
|  | Relative Error | -0.96% | 1.93% | -1.90% | 3.17% | -2.51% |
| **Chengdu-Chongqing** | True Value | -0.19929 | -0.22727 | -0.25615 | -0.21566 | -0.17458 |
|  | Simulation value | -0.1919 | -0.2371 | -0.2608 | -0.2089 | -0.1726 |
|  | Relative Error | -3.70% | 4.35% | 1.83% | -3.15% | -1.12% |
| **Central Plains** | True Value | -0.35586 | -0.27559 | -0.24591 | -0.19176 | -0.17118 |
|  | Simulation value | -0.355 | -0.2738 | -0.2525 | -0.1977 | -0.1723 |
|  | Relative Error | -0.24% | -0.64% | 2.69% | 3.08% | 0.67% |
